# Towards a Semen Proteome of the Dengue Vector Mosquito: Protein Identification and Potential Functions

**DOI:** 10.1371/journal.pntd.0000989

**Published:** 2011-03-15

**Authors:** Laura K. Sirot, Melissa C. Hardstone, Michelle E. H. Helinski, José M. C. Ribeiro, Mari Kimura, Prasit Deewatthanawong, Mariana F. Wolfner, Laura C. Harrington

**Affiliations:** 1 Department of Molecular Biology and Genetics, Cornell University, Ithaca, New York, United States of America; 2 Department of Entomology, Cornell University, Ithaca, New York, United States of America; 3 Laboratory of Malaria and Vector Research, National Institute of Allergy and Infectious Disease, National Institutes of Health, Bethesda, Maryland, United States of America; Mahidol University, Thailand

## Abstract

**Background:**

No commercially licensed vaccine or treatment is available for dengue fever, a potentially lethal infection that impacts millions of lives annually. New tools that target mosquito control may reduce vector populations and break the cycle of dengue transmission. Male mosquito seminal fluid proteins (Sfps) are one such target since these proteins, in aggregate, modulate the reproduction and feeding patterns of the dengue vector, *Aedes aegypti*. As an initial step in identifying new targets for dengue vector control, we sought to identify the suite of proteins that comprise the *Ae. aegypti* ejaculate and determine which are transferred to females during mating.

**Methodology and Principal Findings:**

Using a stable-isotope labeling method coupled with proteomics to distinguish male- and female-derived proteins, we identified Sfps and sperm proteins transferred from males to females. Sfps were distinguished from sperm proteins by comparing the transferred proteins to sperm-enriched samples derived from testes and seminal vesicles. We identified 93 male-derived Sfps and 52 predicted sperm proteins that are transferred to females during mating. The Sfp protein classes we detected suggest roles in protein activation/inactivation, sperm utilization, and ecdysteroidogenesis. We also discovered that several predicted membrane-bound and intracellular proteins are transferred to females in the seminal fluids, supporting the hypothesis that *Ae. aegypti* Sfps are released from the accessory gland cells through apocrine secretion, as occurs in mammals. Many of the *Ae. aegypti* predicted sperm proteins were homologous to *Drosophila melanogaster* sperm proteins, suggesting conservation of their sperm-related function across Diptera.

**Conclusion and Significance:**

This is the first study to directly identify Sfps transferred from male *Ae. aegypti* to females. Our data lay the groundwork for future functional analyses to identify individual seminal proteins that may trigger female post-mating changes (e.g., in feeding patterns and egg production). Therefore, identification of these proteins may lead to new approaches for manipulating the reproductive output and vectorial capacity of *Ae. aegypti*.

## Author Summary

Dengue is a potentially lethal infection that impacts millions of humans annually. This disease is caused by viruses transmitted by infected female *Aedes aegypti* mosquitoes during blood feeding. No commercial vaccine or treatment is available for dengue infection. One way to break the disease transmission cycle is to develop new tools to reduce dengue vector populations. Seminal fluid proteins (Sfps) produced in the reproductive glands of male mosquitoes and transferred to females in the ejaculate during mating could be the target of such a tool. In related insects, Sfps modulate female reproduction and feeding patterns. Here we report 145 proteins that are transferred to females in the *Ae. aegypti* ejaculate. The proteins, which include Sfps and sperm proteins, fall into biochemical classes that suggest important potential roles in mated females. Of particular interest are proteins that could play roles in fertility and hormonal activity (including pathways involved in egg development and utilization of the blood meal). Our results lay important groundwork for new control strategies by identifying candidate proteins that may alter the reproductive biology or blood-feeding patterns of female *Ae. aegypti* and ultimately reduce the global burden of dengue.

## Introduction

Male seminal fluid proteins (Sfps) influence female reproductive and feeding behaviors in a range of insects studied to date (reviewed in [1],[2]). Therefore, these proteins may provide targets or pathways that can be manipulated to reduce pathogen transmission by blood-feeding arthropods. The *Aedes aegypti* mosquito transmits several pathogens of concern to human health, including the viruses that cause dengue and dengue hemorrhagic fever (DHF) ([3]). Dengue, the most important mosquito-borne virus impacting human health, is a re-emerging disease in the tropical regions of the world. There is currently no vaccine against, or cure for, dengue, although research in this area is ongoing ([4]–[6]). Therefore, prevention of dengue infection depends heavily on control of its mosquito vector.

Understanding mosquito reproductive biology is critical to developing effective vector control methods. Previous research on *Ae. aegypti* suggests that mating and, specifically, male-derived proteins may play an important role in modulating female reproduction and feeding behavior. Upon mating, female *Ae. aegypti* undergo a series of time-dependent behavioral and physiological changes. Relative to virgin females, mated females have increased egg development and oviposition rates ([7],[8]), blood digestion rates ([9],[10]), and blood meal size ([10]). Mated females also have a lower likelihood of being inseminated by another male ([11]), of flying ([12],[13]), and of responding to host cues ([14]–[17]), and they have a reduced daily blood-feeding frequency ([18]). These changes in mated females appear to be induced by molecules produced in males' accessory glands (AG) and transferred to the female during mating ([9],[19]–[28]). In two other Dipteran species, individual AG-derived Sfps have been associated with functions in mated females. In *Drosophila melanogaster*, experimental studies have demonstrated that specific AG-derived Sfps influence a wide range of female post-mating behaviors including oogenesis (sex peptide), ovulation (ovulin), sperm storage and release from storage (Acp36DE, Acp29AB, CG1652/CG1656, CG17575, CG9997), propensity to re-mate (sex peptide), activity level (sex peptide), and feeding (sex peptide; reviewed in [2],[29]–[32]). In *Anopheles gambiae*, transglutaminase derived from male reproductive glands is necessary for the formation of the mating plug which is required for proper sperm storage ([33]). In both *Ae. aegypti* and *D. melanogaster*, secretions from the male AGs are necessary for fertility ([19],[34]). Identification of individual bioactive Sfps causing post-mating changes in female *Ae. aegypti* has not yet been accomplished and is a long-term goal of our research.

Previously, we identified over 250 proteins from male *Ae. aegypti* reproductive glands (AGs and seminal vesicles; [35]; L. Sirot, M. Wolfner, L. Harrington, unpubl. data). Fifty-three of those proteins were considered to be putative Sfps based on the criteria that they contained predicted secretion signal sequences and were not known to be housekeeping or structural proteins ([35]). However, we did not have direct evidence that those proteins were transferred to females during mating. In the current study, we identify a suite of proteins that are transferred from males to females during mating, and are thus candidate regulators of female behavior and physiology. Male- and female-derived proteins in the female reproductive tract were distinguished using an approach adapted from a study in *D. melanogaster* ([36]) that combined proteomics with a stable-isotope labeling technique (using ^15^N). We adapted this method to blood-feeding mosquitoes and discovered a set of proteins transferred from male to female *Ae. aegypti*. Among the Sfps we identified are potential modulators of protein activation/inactivation, sperm utilization, and ecdysteroidogenesis. Few of the *Ae. aegypti* Sfps we detected are homologs of known or predicted Sfps in other insect species, although many are in protein classes that are conserved across seminal fluid of a wide range of taxa ([37],[38]). Furthermore, our finding of intracellular and membrane-bound proteins in the transferred Sfps supports the hypothesis that *Ae. aegypti* Sfps are secreted, at least in part, through apocrine processes (pinching off of the apical portion of the cells into vesicles containing Sfps; [39]) in the accessory glands.

In the process of identifying the Sfps, we also identified a subset of 52 putative *Ae. aegypti* sperm proteins. The *D. melanogaster* homologs of many of the predicted *Ae. aegypti* sperm proteins are also sperm proteins ([40]), suggesting conservation of sperm-related function across Diptera. Some of the proteins we have identified may be useful targets for control of *Ae. aegypti* and may be applicable to other mosquito vectors.

## Methods

### ^15^N-Labeling of female *Ae. aegypti* proteins

#### Overview

In order to distinguish male- and female-derived proteins in the mated female reproductive tract, we adapted a stable-isotope labeling technique that had been developed for *D. melanogaster* ([36]). Stable-isotope labeling of proteins makes their peptides unidentifiable by standard mass spectrometry analysis. Therefore, we mated labeled females to unlabeled males in order to identify male-derived proteins in the female reproductive tract. The steps involved in this process (described in detail below) are: stable-isotope labeling of yeast as food for mosquito larvae, rearing of mosquitoes on labeled yeast, mating of labeled females to unlabeled males, extraction of proteins from mated female reproductive tracts, and mass spectrometric analysis of the extracted proteins.

#### Yeast labeling

*Saccharomyces cerevisiae* strain D273-10B was used for all experiments. Two batches of yeast were prepared: one labeled with ^15^N and one unlabeled. Isotopic labeling was performed following the methods of Findlay et al. [36] with adjustments for incorporating the label into mosquitoes. Briefly, yeast was grown to saturation (∼16 h at 30°C) in 200 mL of minimal medium consisting of 20 g of glucose, 1.7 g of yeast nitrogen base without amino acids and either 5 g of ^15^N labeled ammonium sulfate (>99% ^15^N-enrichment; Cambridge Stable Isotopes, Andover MA, USA) or 5 g of unlabeled ammonium sulfate, in sterile water. The following day, 800 mL of additional medium was added and the yeast was grown for another 24 h. Yeast was harvested by centrifugation at 5,000 rpm at 4°C for 10 min, and the pellet was re-suspended in 30 mL of sterile water and centrifuged such that 15 mL of excess water was removed to yield a final volume of 15 mL of yeast slurry. The yeast slurry was stored at 4°C for less than two weeks before use.

#### Mosquito rearing

The Liverpool strain of *Aedes aegypti* L. was used for all experiments. Initially, eggs were hatched under vacuum and 200 first instar larvae were transferred to shallow pans containing 1 L of sterile water. Approximately 1 mL of the yeast slurry was added to each larval rearing pan every 1–2 days until pupation occurred. Preliminary data showed that females from the ^15^N-labeled treatment required additional nutrition beyond the yeast slurry (as assessed by their inability to fly and, thus, mate). Therefore, we supplemented the rearing pans with 200 mL of inoculum of rearing-water from a previous cohort of larvae grown under the same treatment (*i.e.*, unlabeled or ^15^N-labeled yeast). The resulting larvae produced females that were able to fly and mated readily. Unlabeled males from the same mosquito strain were reared in pans of 200 larvae/1 L water and fed a 1∶1 mixture of Brewer's yeast and lactalbumin until pupation. All pupae were placed individually into vials and held for emergence. All adult mosquitoes were maintained on 20% sucrose solution on soaked cotton wicks. Reproductive tract samples were dissected from unlabeled and ^15^N-labeled virgin females to test the effectiveness of the labeling technique.

#### Mating

^15^N-labeled virgin females (3–5 days after emergence) were individually introduced into a 5 L bucket container that contained 20–40 unlabeled males (4–6 days after emergence). If a successful mating event was observed, both the female and male were removed from the bucket at the termination of mating as the pair began to separate. After mating, each female was placed individually in a test-tube and stored on ice for dissection. Females that did not mate within approximately 5 min were removed from the male cage and discarded; a new female was introduced into the male cage for mating. Dissections of the ^15^N-labeled females mated to unlabeled males were conducted within 30 min of mating.

#### Dissections

Female lower reproductive tracts (i.e., spermathecae, bursa, common oviduct) were dissected in MOPS buffer (80 mM NaCl, 10 mM KCl, 1 mM CaCl_2_, 0.2 mM MgCl_2_,10 mM MOPS) on ice and homogenized in 20 µl Dulbecco's PBS (DPBS) with protease inhibitors (Roche Complete Protease Inhibitor Tablets, Indianapolis, IN). Reproductive tracts from 20 females were pooled for each biological replicate. Samples were centrifuged at 12,000 rpm for 30 min at 4°C. The supernatant was transferred to a separate tube, the pellet was resuspended in 20 µl DPBS with protease inhibitors, and 20 µl of 2× SDS sample buffer (125 mM Tris–HCl pH 6.8, 20% glycerol, 4% SDS, 10% β-mercaptoethanol, 0.001% bromophenol blue) was added to each sample. The samples were boiled for 4 min and stored at −80°C.

#### Identification of proteins in sperm derived from seminal vesicle and testes

In order to distinguish Sfps from sperm proteins, we conducted proteomic analyses of sperm-enriched samples derived from the seminal vesicles or the testes. Seminal vesicles or testes from 40 males were dissected in DPBS with protease inhibitors, on ice. The tissues were then placed in a fresh droplet of DPBS, teased apart with a needle, swirled in the buffer to release sperm, and then removed from the droplet. The buffer droplet containing the sperm was transferred to a microcentrifuge tube containing 500 µl DPBST (DPBS with 0.1% Tween-20). The samples were spun at 20,800×g for 5 min at 4°C. The supernatant was discarded and the pellet was washed twice in DPBST. After the final wash, the pellet was resuspended in 2× SDS sample buffer. We considered the resulting samples as “sperm-enriched” as they contained not only sperm, but likely also some tissue, tissue secretions, and Sfps.

#### Protein separation and identification

Both the supernatant and the pellet samples were analyzed using two independent biological replicates of each sample type, except for the virgin females for which only one sample each was used for verification of our techniques. Protein separation and identification was conducted as previously described [35]. Briefly, proteins from each sample were separated by electrophoresis on a one-dimensional 5–15% gradient SDS polyacrylamide gel and visualized using Simply-Blue SafeStain (Invitrogen, Carlsbad, CA). The Cornell University Life Sciences Core Proteomics and Mass Spectrometry facility conducted in-gel digestion, tryptic peptide extractions, and Nano-LC-MS/MS. The nanoLC was carried out using an LC Packings Ultimate integrated capillary HPLC system equipped with a Switchos valve switching unit (Dionex, Sunnyvale, CA), which was connected in-line to a hybrid triple quadropole linear ion trap mass spectrometer, 4000 Q Trap (ABI/MDS Sciex, Framingham, MA) equipped with Micro Ion Spray Head ion source. The resulting MS and MS/MS data were submitted for database searching using the MASCOT search engine version 2.3 (Matrix Science, Inc., Boston, MA) or ProteinPilot software 1.0 (Applied Biosystems, Foster City, CA) against three databases (see “Databases” section below): the *Ae. aegypti* predicted peptide (AaegL1.2 Gene Build; hereafter “Vectorbase”) database (including supplemental peptides from AaegL 1.1 Gene Build, http://aaegypti.vectorbase.org/index.php; MASCOT program, Matrix Science, Boston, MA), a 6-frame translation of the *Aedes* genome (versionAaegL1.2; MASCOT program; hereafter “6-frame translation”), and a database of small (<150 amino acid) predicted peptides (ProteinPilot; hereafter “small peptides”). The default search settings used for protein identification were: one mis-cleavage for full trypsin with variable carbamidomethyl modification of cysteine, and a methionine oxidation. For the searches using MASCOT, the false discovery rate (FDR) was estimated for a measure of random identification from the same database. To estimate the FDR, an automatic decoy database search was performed in which a database of random sequences was generated and tested for raw spectra along with the real database.

Protein identifications were based on a significance threshold of <0.05. Additionally, we only considered proteins to be high confidence hits if either two different peptides from the same sample exceeded the significance threshold or if one peptide hit exceeded the significance threshold in two independent biological replicates (single or multiple peptide hits are reported in Tables S1 and S2). Hits from the 6-frame translation and the small peptides database were compared to the Vectorbase database using BLASTP, and queries with a significant match were removed. Any remaining hits from the 6-frame translation were searched for a predicted gene using the GeneID 1.2 prediction program (http://genome.crg.es/geneid.html) and only these hits were used in further analyses. We tested for predicted secretion signal sequences using SignalP 3.0 (www.cbs.dtu.dk/services/SignalP/, [41]) and for predicted protein domains using SMART (http://smart.embl-heidelberg.de/, [42]) and Pfam (http://pfam.sanger.ac.uk/, [43]). The relative quantitation of identified proteins in each biological sample was estimated using the exponentially modified protein abundance index (emPAI, [44]) and are reported in Tables S1 and S2.

#### Databases

All of the databases are derived from sequencing of the Liverpool strain. The Vectorbase database is based on 8× coverage and includes 4,758 supercontigs, 1.3 gigabases, 15,988 genes, and 17, 402 predicted peptides (http://www.vectorbase.org/Aedes_aegypti/Info/Index). To obtain the 6-frame translation database, the *Ae. aegypti* contigs were downloaded from Vectorbase (version AaegL1.2, September 2009). A program written in Visual Basic 6.0 by one of the authors (JMCR) obtained the 6-frame translation using the eukaryotic codon table. Sequences between stop codons were written to a fasta file retaining the frame and contig coordinates. This database includes 81,550,487 sequences. The small peptides database was generated by one of the authors (JMCR) through *ab initio* gene prediction based on the AaegL1.1 release using the GeneID prediction program (http://genome.crg.es/software/geneid/, [45]) and includes 24,092 sequences.

#### Identification of homologs to *Ae. aegypti* Sfp and sperm protein-encoding genes

By using BLASTP, we identified homologs of the *Ae. aegypti* proteins in the genomes of three other Diptera (*Culex quinquefasciatus*, *Anopheles gambiae*, and *D. melanogaster*) based on amino acid similarity. The divergence time between *Aedes* and *Drosophila* is predicted to be 250 million years ago ([46]). The *Anopheles* and *Aedes* genera are predicted to have diverged approximately 226 million years ago ([47]). Divergence time for *Aedes* and *Culex* is more recent than the divergence between *Aedes* and *Anopheles* ([47]). For the proteins identified from the *Ae. aegypti* Gene Build database, we defined homologs as reciprocal best BLASTP hits with ≥30% identity and E values≤10^−3^. For proteins identified from the Supplemental predicted peptides, the 6-frame translation and the small peptides databases, we used unidirectional BLASTP searches of the *Ae. aegypti* hits against the databases from the other three species.

## Results and Discussion

### Isotope labeling technique

In order to identify male-derived proteins that are transferred to females during mating, we used a whole-organism isotope labeling method. The principle of this method is to mate males to females whose proteins are labeled with the stable isotopes so as to exclude the female proteins from proteomic identification. Specifically, female proteins are labeled with ^15^N, which shifts their masses upward such that the masses of female-derived peptides do not match those expected in a standard search (uncorrected for ^15^N) of a predicted protein database. The method was developed by Krijgsveld et al. [48] and first used to identify Sfps by Findlay et al. [36] in *D. melanogaster*. We adapted this method to label female-derived proteins in *Ae. aegypti*. As with *D. melanogaster*, we reared larvae on yeast whose only nitrogen source was ^15^N. However, in order to generate females that could fly and mate, we had to supplement the larvae with an inoculum of rearing-water from larvae previously reared on ^15^N-labeled yeast (see Methods for more detail). To verify that the ^15^N-labeling technique sufficiently labeled female-derived proteins, we conducted nanoLC-MS/MS on protein samples from the reproductive tracts of labeled and unlabeled virgin females. We initially analyzed protein samples from two arbitrarily-chosen molecular weight ranges (∼30 kD to 50 kD and ∼98 kD to 120 kD) of both types of females. Using the Vectorbase database, we identified 115 proteins from the unlabeled female samples (Table S3) and no proteins from the labeled female samples. To further verify the labeling technique, we conducted nanoLC-MS/MS on the remaining gel sections from the labeled virgin female sample. We did not identify proteins from any of these samples. These results demonstrate that any proteins we identify in the reproductive tracts of labeled females mated to unlabeled males are highly likely to be male-derived. Furthermore, the technique we developed for *in vivo* stable-isotope labeling of *Ae. aegypti* proteins could be applied to other studies (e.g., to quantify proteomic changes in females in response to mating and/or blood-feeding or to distinguish mosquito-derived proteins from those of their pathogens, parasites, and/or endosymbionts; [49]).

### Proteins transferred to females during mating

In our search against the Vectorbase database, we identified 128 proteins in the reproductive tracts of labeled females after mating with unlabeled males (Tables 1 and 2). Since the *Ae. aegypti* genome sequence is relatively new and thus the current annotation might still be missing actual genes, we also searched our mass spectrometry results against a 6- frame translation database of the *Ae. aegypti* contigs (Version AaegL1.2) and against a database of predicted small peptides (<150 amino acids). In our search against the 6-frame translation, we identified 12 novel predicted semen proteins (Tables 1 and 2). We identified 5 novel predicted small peptides from the small peptides database (Tables 1 and 2). The sequences of the unannotated predicted proteins and peptides are provided in Table S4. Thus, in total, we identified 145 male-derived predicted proteins that are transferred during mating to females. These proteins include Sfps and sperm proteins. For all searches, the FDR was ≤1%.

**Table 1:** Predicted seminal fluid proteins transferred in *Aedes aegypti* ejaculate.

Molecular function	Predicted protein class	*Aa* a	Molecular function (cont.)	Predicted protein class	*Aa*
**Binding**	Annexin	11302	**Proteolysis/ Catalysis (cont.)**	Protease	01588
	Calcyphosine	08489			02000
	Dipeptidase	08893			06403
	Fibrinogen	01713			06414
	Kakapo	02829			06421
	Lectin	Supp4872			06429
		04679			10725
	Mitochondrial brown fat uncoupling protein	07046			11558
	Moesin	07915			12217
	Mucin	00718			15386
	Odorant-binding	AaegSfp1		Protease inhibitor	02715
	Phosphatidylethanol- binding protein	11263			AaegSfp2
					AaegSfp3
	Tubulin β-chain	02848		Thioesterase	03569
	None	00479		Transferase	03746
		08274		None	02793
		09201			13559
**Oxidoreductase**	Catalase	13407			17451
	Decarboxylase	05790			17460
	Dehydrogenase	04338	**Structural**	Actin	01928
		05308			05964
		06928		Vitellogenin	05815
		10464		None	03348
		12014	**Transport**	Cytochrome c oxidase subunit	00929
	NADH-ubiquinone oxidoreductase subunit	05946			13751
	Peroxidase	04112		Glutamate receptor	09813
**Proteolysis/ Catalysis**	Aminopeptidase	02399		Mitochondrial glutamate carrier	11276
		02978		Sodium/calcium exchanger	12480
		07201	**Other**	Niemman-Pick Type C-2	09760
	Asparaginase	02796		Rab GDP-dissociation inhibitor	12904
	ATP synthase subunit	06516		Venom allergen	09239
		07777		None	04944
		11025			05219
		12035			10824
					Supp3543
		12819			Supp4095
	ATPase	14053			AaegSfp5
	Dehydrogenase	04294			AaegSfp6
	Dynein	11478			AaegSfp7
	Gamma glutamyl transpeptidase	10935			AaegSfp8
		14580			AaegSfp9
	Glutathione transferase	11741			AaegSfp10
	Hydrolase	03666			AaegSfp11
		06485			AaegSfp12
	Kinase	12359			AaegSfp13
		12731			AaegSfp14
	Lipase	07063			
	Mannosidase	05763			

a 5-digit numbers are the Vectorbase database identification numbers without the proceeding “AAEL0”. Numbers with “Supp” prefix refer to proteins from the Supplementary predicted peptide database from AaegL1.1 Gene Build. Numbers with the prefix “AaegSfp” refer to proteins from either the 6-frame translation or the small peptides databases. The amino acid sequences for all of the “Supp” and “AaegSfp” predicted proteins are given in Table S4.

**Table 2:** Predicted sperm proteins transferred in *Aedes aegypti* ejaculate.

Molecular function	Predicted protein class	*Aa* a	Molecular function (cont.)	Predicted protein class	*Aa*
**Binding**	Aminopeptidase	06975	**Proteolysis/ Catalysis (cont.)**	Protease inhibitor	AaegSp1
	Heat shock protein 70	Supp4130		None	06509
	Histone	00490			10754
		15674			17349
	Reticulocalbin	14589	**Structural**	Actin	01673
	Tubulin α-chain	06642			11197
		13229		Myosin	12543
	None	00637		Tubulin β-chain	02851
		08779			05052
		10149	**Transport**	ADP, ATP carrier	04855
		10882		Cytochrome c	04457
		14231		Cytochrome c oxidase subunit	05170
**Oxido-reductase**	Dehydrogenase	00454		Ubiquinol-cytochrome c reductase unit	03675
		02881			05269
		03757		Voltage-dependent anion-selective channel	01872
		08166		None	17508
	NADH-ubiquinone oxidoreductase	12552	**Other**	Netrin receptor	07195
**Proteolysis/Catalysis**	Aconitase	03734		None	09707
	Aminopeptidase	00108			12282
	ATP synthase subunit	02827			17096
		05173			Supp4104
		05610			Supp7141
		05798			AaegSp2
		08787			AaegSp3
		08848			
		12175			
	Kinase	06042			
	Protease	03308			

a 5-digit numbers are the Vectorbase database identification numbers without the proceeding “AAEL0”. Numbers with “Supp” prefix refer to proteins from the Supplementary predicted peptide database from AaegL1.1 Gene Build. Numbers with the prefix “AaegSp” refer to proteins from either the 6-frame translation or the small peptides databases. The amino acid sequences for all of the “Supp” and “AaegSp” predicted proteins are given in Table S4.

Nine of the identified proteins share identical amino acid sequence with other *Ae. aegypti* predicted proteins in the regions that were identified by our mass spectrometry analysis. As a result, we cannot distinguish amongst these proteins in our samples. For simplicity, we have listed just one protein from each of these pairs or groups of indistinguishable proteins in Tables 1 and 2. However, we list the identities of all proteins in these pairs or groups in Table S5.

### Comparison to previously identified *Ae. aegypti* reproductive gland proteins

Of the 145 transferred proteins we identified using the whole-organism isotope labeling method, 123 are newly-recognized components of *Ae. aegypti* semen. The remaining 22 were previously identified as putative Sfps ([35]), and we demonstrate here that they are transferred to females during mating. Thirty-one additional proteins were identified as putative Sfps in our previous study ([35]), but we did not detect them as transferred in our current study. Those proteins may not be transferred to females, or may be transferred at quantities below our detection threshold or with post-translational modifications that render them unidentifiable by standard mass spectrometry. Of the 123 newly-recognized seminal proteins, 84 were previously identified from the reproductive glands of *Ae. aegypti*, however they were not designated as putative Sfps because they lacked predicted secretion signal sequences ([35]; L. Sirot, M. Wolfner, L. Harrington, unpubl. data).

### Distinguishing seminal fluid proteins from sperm proteins

In order to distinguish Sfps from sperm proteins among those transferred to females, we conducted a proteomics analysis of sperm-enriched samples from the seminal vesicles (SVs) and testes of virgin males. Sperm-enriched samples were obtained by releasing sperm from these organs, pelleting the sperm by centrifugation, and washing them repeatedly, as in Dorus *et al.* [40] (see Methods for details). We found 101 proteins that overlapped between our sperm-enriched samples from seminal vesicles and our sperm-enriched samples from testes. Of these 101 putative sperm proteins, 52 were detected as transferred to females during mating, providing a high-confidence subset of putative *Ae. aegypti* sperm proteins (Table 2).

Of the 145 total transferred proteins (see section “Proteins transferred to females during mating”), 16 were isolated from only one of the sperm-enriched tissues (SV: 5; testes: 11) and therefore were considered SV- or testes-derived Sfps, respectively, although we recognize that these could be sperm proteins. Additionally, 77 of the transferred proteins did not overlap with either sperm-enriched sample. Together, the 5 SV-derived Sfps, 11 testes-derived Sfps, and 77 of the 145 total transferred proteins that did not overlap with the sperm-enriched samples comprise a total of 93 proteins assigned with high-confidence as *Ae. aegypti* Sfps (Table 1).

### Seminal fluid proteins

**Figure 1:**
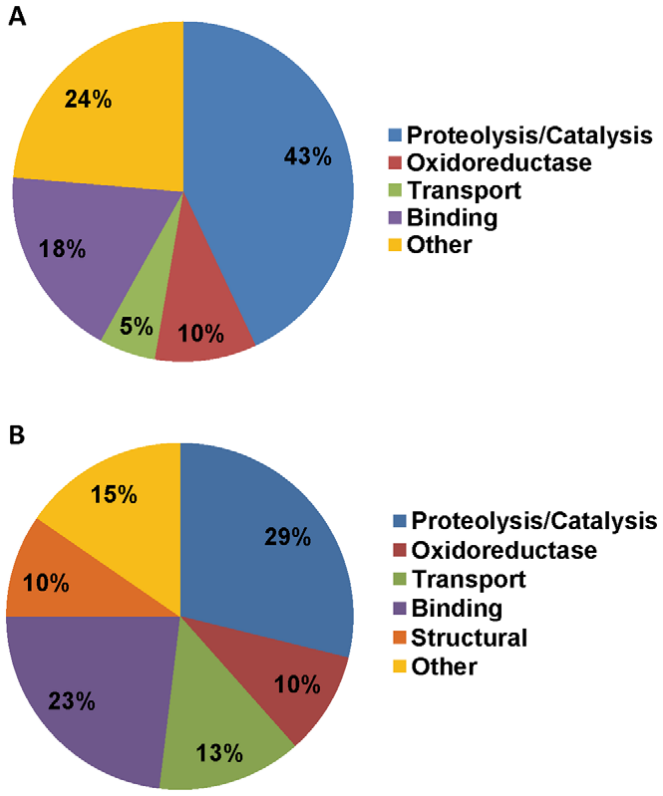
Gene Ontology molecular function categories of *Aedes aegypti* seminal fluid and putative sperm proteins. A. Seminal fluid proteins; B. Putative sperm proteins.

The *Ae. aegypti* Sfps identified represent a wide-range of predicted protein classes including proteolysis regulators, lectins, lipases, oxidoreductases, a cysteine-rich secretory protein (CRISP) and a venom allergen, and fall into a variety of Gene Ontology predicted molecular function classes (Table 1; Fig. 1A). Unlike Sfps in *Drosophila* ([31],[36],[50]) and *An. gambiae* ([33],[51]), in which some groups of Sfps tend to be spatially clustered, we found little evidence for spatial clustering of the 93 Sfp genes in the *Ae. aegypti* genome, with one exception. Four Sfp genes (AAEL006403; AAEL006414; AAEL006421; AAEL006429) clustered within a 23 kB region on supercontig 1.204. One gene in this region (AAEL006430) encodes a protein that was not detected in the present study and may either not be transferred or may be transferred at a level that was undetectable by our methods. The proteins encoded by all five of the genes in this region have predicted trypsin domains and their shared amino acid sequence identities range from 36 to 64%.

As might be expected, the genes encoding the Sfps identified in this study tend to have highly male-biased expression when gene expression of whole males is compared to gene expression of whole females ([52]). Half (26 of 51) of the genes for which microarray data are available have significantly higher expression (at P≤0.001) in males than in non-blood-fed females (O. Marinotti, pers. comm.), as compared to a genome average of 16% (Chi-square test; *X^2^_1_* = 47.7; P<0.001; [52]). By comparison, 63% of the *D. melanogaster* Sfps identified by [36] have significantly higher expression (at P≤0.01) in males than in females as compared to a genome average of 12% (*X^2^_1_* = 345.9; P<0.001; [53]). Surprisingly, transcript levels of two *Ae. aegypti* Sfp-encoding genes (encoding a predicted kinase, AAEL012359, and a predicted zinc metalloprotease, AAEL012217) are significantly higher in non-blood-fed females than in males.

#### Sequence comparisons to other Diptera

Table S1 shows the extent to which homologs of the 93 putative Sfps can be detected in three other Dipteran genomes (*Cx. quinquefasciatus*, *An. gambiae*, and *D. melanogaster*). In comparison to known or predicted Sfp- or male AG-encoding genes from *An. gambiae* or *D. melanogaster*, *Ae. aegypti* Sfp genes generally had little sequence similarity. None of the *An. gambiae* homologs to *Ae. aegypti* Sfps is among the previously-identified *An. gambiae* AG genes ([33],[51]) or mating plug protein-encoding genes ([33]). Only two *D. melanogaster* (CG31704, and CG5162) homologs to *Ae. aegypti* Sfps (AaegSfp3 and AAEL005815, respectively; range of percent identity: 41–59%) are among the known *D. melanogaster* Sfps or AG genes (Table S1; [31],[31], [36],[54]). Additionally, three *Ae. aegypti* Sfps (AAEL005815; AAEL004112; AAEL000718) share sequence similarity (range: 30–84% identity) with four of the 13 AG genes identified from the Mediterranean fruit fly, *Ceratitis capitata* (clones 11c, 18a, 30c, 33a; [55]).

Below, we discuss (i) the unannotated, newly-identified predicted proteins from the 6-frame translation and small peptides database, (ii) new insights into the mode of *Ae. aegypti* Sfp secretion from Vectorbase identified proteins, and (iii) the potential biological functions of a subset of Sfps in modulating reproductive and physiological processes within the mated female. In our previous report on *Ae. aegypti* Sfps ([35]), we discussed the potential roles of these proteins in a number of processes including protein folding, antimicrobial activity, and sperm utilization by females. Although the proteins we have identified in the current report include many in the same classes and predicted functions as our previous report (and include 22 of the same proteins), we have specifically chosen to highlight proteins that suggest functions in the mated female that were not discussed in our previous report ([35]). These protein classes are not necessarily the most highly represented of the predicted Sfps.

#### Unannotated seminal fluid proteins

Based on comparisons to the 6-frame translation and small peptides databases, we discovered 14 previously unannotated predicted Sfps (Table 1). Of the 9 predicted proteins from the 6-frame translation, only one (AaegSfp2) had a significant hit to the SMART or PFAM database. This protein includes a predicted secretion signal sequence and a Kazal-type serine protease inhibitor domain. Interestingly, another predicted secreted protein of the 9 hits (AaegSfp8) shared a high degree of sequence similarity (70% identity; E value = 2e^−74^) with one of the Vectorbase predicted proteins, AAEL010824. AAEL010824 and AaegSfp8 are located on the same contig within 34 Kb of each other. Two of the other hits from the 6-frame database also had predicted secretion signal sequences. Of the 5 hits to the small peptides database, we found one predicted secreted Kazal-type serine protease inhibitor (AaegSfp3), one predicted secreted odorant-binding protein (AaegSfp1), and three hits with no predicted secretion signal sequence and no predicted protein domains.

#### Mode of secretion

Sixty-two of the Sfps that we identified are predicted intracellular or membrane-bound proteins (e.g., ATPases, dipeptidyl peptidase, gamma glutamyl transpeptidase, glutathione S-transferase, angiotensin converting enzyme). Predicted intracellular proteins also have been reported in the seminal fluid of other organisms including bed bugs ([56]), honey bees ([57]), and humans ([58], [59]), and in the AGs and seminal fluids of *D. melanogaster* ([60]; G. Findlay & W. Swanson, pers. comm.). In bed bugs, Sfps of predicted intracellular origin have been considered potential cell contaminants ([56]); whereas, in honeybees, it has been suggested that these proteins may be secreted through non-standard secretion routes ([57]). In *Ae. aegypti*, our finding of intracellular and membrane-bound Sfps is consistent with the hypothesized modes of secretion in the AGs of this species: based on cytological studies using light and electron microscopy, cells in the anterior portion of the glands are thought to secrete proteins by pinching off apically (“apocrine secretion”; [39],[61]), whereas cells in the posterior portion of the glands are thought to secrete proteins through granules and/or via rupture of the cell membrane (“holocrine secretion”; [61],[62]; but see [39]). Inside the female reproductive tract, the ejaculate contains “vacuoles” that grow over time after mating and then disappear by 24 h post-mating ([62]). Four of the Sfps we identified are subunits of the membrane-bound vacuolar-type proton ATPase and thus may be part of these vacuoles. Future studies could investigate whether the male-derived vacuolar ATPase is functional in the ejaculate vacuole membrane in the female reproductive tract and therefore may play a role in regulating the release of the contents of the vacuoles.

Importantly, our results emphasize that studies of Sfps in other species should not exclude proteins whose sequence (alone) suggest that they are intracellular and membrane-bound proteins. For species in which intracellular and/or membrane-bound proteins are found in the seminal fluid (e.g., bed bugs and honey bees), further research should be conducted on the mode of secretion of the male reproductive gland cells. Furthermore, secretion by *Ae. aegypti* accessory glands could serve as a model for secretion in systems such as the male reproductive glands of mammals, since mammalian prostasomes, exosomes, and epididymisomes contain many of the same classes as found among *Ae. aegypti* Sfps ([63]).

#### Proteolysis

Across a wide range of organisms, seminal fluid is rich in proteolysis regulators (e.g., [37],[38],[64]–[67]). *Ae. aegypti* is no exception. Thirteen of the 93 Sfps we identified in *Ae.aegypti* are predicted regulators of proteolysis. These include predicted trypsins, a zinc carboxypeptidase, a metalloprotease, a serine protease inhibitor (serpin), an angiotensin converting enzyme, and a proprotein convertase in the subtilisin/kexin type 4 (PCSK4) family (Tables 1 and S1). The predicted or known functions of seminal fluid proteolysis regulators include regulating the liquefaction of semen and/or mucus in the female reproductive tract ([68],[69]); protection of sperm from premature acrosome reactions ([67]); and activation and/or degradation of other reproductive proteins ([70]). We have previously discussed the potential role of predicted proteolysis regulators in *Ae. aegypti* seminal fluid ([35]). Here, we highlight the novel finding of a predicted proprotein convertase subtilisin/kexin type 4 (PCSK4) protein in insect seminal fluid.

PCSK4s can activate precursors of membrane receptors, peptide hormones, antibacterial peptides and neuropeptides through proteolytic processing ([71],[72]). Interestingly, the predicted PCSK4 (AAEL010725) found in this study shares close sequence similarity (56% identity; E-value: 2e-158) with the *Ae. aegypti* protein that processes vitellogenin (AAEL003652; [72]). To our knowledge, proprotein convertases have not been reported previously in insect seminal fluid. Transcripts of the *D. melanogaster* gene encoding one protein in this class (CG10702) are enriched (relative to whole body) in the AGs ([73]). The protein they encode contains a predicted secretion signal sequence (SignalP; [41]), but it has not yet been detected in the seminal fluid in this species ([36]).

#### Steroidogenesis

One of the Sfps we identified (AAEL009760) is a predicted sterol carrier in the Niemann-Pick type C-2 (NPC2) family. In insects, sterol carriers are essential for the production of ecdysteroids (ECDs) (e.g., [74]). ECDs are hormones that influence molting, gametogenesis, vitellogenesis, and other reproductive processes (reviewed in [75],[76]). In *Ae. aegypti*, ECDs are essential components of a signaling cascade linking blood meal intake with vitellogenesis (e.g., [77]; reviewed in [78], [79]). Although a role for the male-derived NCP2-like protein has not yet been determined in *Ae. aegypti*, findings in other Dipteran species suggest that male AGs can not only synthesize ECD (*An. gambiae*, [80]) but also stimulate ECD production in females (*D. melanogaster*, [81]). We suggest that AAEL009760 could potentially contribute to the regulation of ECD biosynthesis in *Ae. aegypti* male reproductive tracts and/or in mated females.

### Sperm and sperm-associated proteins

From *Ae. aegypti* sperm-enriched tissue samples, we identified 101 sperm or sperm-associated proteins. Fifty-two were found among proteins transferred to females during mating (Table 2); the remaining 49 proteins were not detected as transferred (Table S6). These latter 49 proteins included 7 that have homologs found in the *D. melanogaster* sperm proteome ([40]). It is possible that some of these 49 *Ae. aegypti* proteins are components of somatic cells of the testis and/or SV tissues and play a role in spermatogenesis or sperm maintenance, whereas others could be sperm proteins whose abundance was too low for us to detect in the transferred samples or whose post-translational modifications rendered them unidentifiable by standard mass spectrometry. In the remainder of this section, we will only discuss the 52 proteins detected as transferred (hereafter referred to as “putative sperm proteins”).

#### Sequence comparisons to other Diptera

Table S2 shows the extent to which homologs of the 52 putative sperm proteins can be detected in three other Dipteran genomes (*Cx. quinquefasciatus*, *An. gambiae*, and *D. melanogaster*). Of the 30 proteins with *D. melanogaster* homologs, 17 (57%) of the homologs were found among the identified sperm proteins of *D. melanogaster* ([40]). This level of homology between *Ae. aegypti* putative sperm proteins and *D. melanogaster* sperm proteins suggests that the sperm-related functions of these proteins are conserved between the two species. Non-sperm *D. melanogaster* homologs of *Ae. aegypti* putative sperm proteins may also serve reproductive functions, as suggested by gene expression patterns. Of the 13 *D. melanogaster* homologs not found in the *D. melanogaster* sperm proteome (Table S2), transcripts of 5 are enriched in male and female reproductive tissues, transcripts of 1 are enriched only in male reproductive tissues, and transcripts of 6 are enriched only in female reproductive tissues ([73]).

#### Unnannotated sperm proteins

We discovered three previously unannotated predicted sperm proteins from the 6-frame translation database (Table 2). One predicted protein from the 6-frame translation, AaegSp1, contained a predicted secretion signal sequence and a Kazal serine protease inhibitor domain. The other two hits contained no predicted secretion signal sequence and no conserved protein domains.

#### Potential functions

The likely biological functions of putative *Ae. aegypti* sperm proteins include spermatogenesis, serving as structural components of mature sperm, and sperm locomotion (Fig. 1B). Spermatogenesis-related proteins that we found among *Ae. aegypti* putative sperm proteins include the heat shock protein 70, actin, and tubulin (α- and β-chains). *Ae. aegypti* putative sperm proteins that might contribute to sperm structure or motility include actin, tubulins, dyneins, ATP synthases, protein kinases and kinesin motor proteins ([82]–[86]). *Ae. aegypti* putative sperm proteins that are predicted mitochondrial enzymes include malate dehydrogenase, aconitase, cytochrome c oxidase, and ubiquinol-cytochrome c reductase. In other animals, these proteins generate the energy necessary for sperm locomotion via oxidative phosphorylation and the citric acid cycle ([87]–[92]).

### Summary and conclusion

Secretions of the reproductive glands of male *Ae. aegypti* have previously been shown to induce post-mating changes in female reproductive and feeding behavior ([22],[23],[26]). In order to lay the groundwork for identifying specific proteins causing these effects, we report here 145 male-derived proteins that are transferred to females during mating in *Ae. aegypti*. We distinguished 93 seminal fluid proteins from 52 predicted sperm proteins, thus contributing to the growing understanding of insect ejaculate proteomes ([33],[35],[36],[40],[57],[88],[93]–[95]). Twenty-two of these proteins were previously identified as male reproductive gland proteins ([35]), and we demonstrate here that they are transferred to the female.

The Sfps identified in this study suggest roles in protein activation/inactivation, ecdysteroidogenesis, and sperm utilization. Furthermore, our discovery that many predicted intracellular and membrane-bound proteins are transferred to females in the seminal fluid indicates that findings of such proteins in the seminal fluid of other species (e.g., [56],[57]) may also result from apocrine and/or holocrine secretion from the male reproductive glands ([39],[61]). The putative sperm proteins of *Ae. aegypti* show sequence conservation within Diptera and 17 of their *D. melanogaster* homologs are sperm proteins in that species ([40]) indicating potential conservation of sperm-related functions.

Genes encoding Sfps showed higher male-biased expression than the genome average. On the one hand, this is not unexpected because Sfps are made in the male reproductive tract and are then transferred to females. On the other hand, it is not necessarily predicted *a priori* that Sfp-encoding genes will be male-biased in their expression, and the way we identified the proteins was without bias regarding their genes' expression. That 49% of the *Ae. aegypti* Sfp-encoding genes for which there are microarray data are not male-biased in expression will be important to bear in mind in designing future screens for Sfps.

Together, our results provide a foundation for functional analyses to associate individual Sfps with their function in the mated female. Once functions are identified for individual proteins, investigations of the pathways by which they induce effects on male and female reproductive biology could identify novel targets for control of *Ae. aegypti* and dengue transmission. Of particular interest is to determine how specific Sfps modulate female behavior and physiology (e.g., egg production and blood feeding) and to investigate candidate genes which increase the reproductive success of male *Ae. aegypti* that are to be used in genetic control strategies.

## Supporting Information

Table S1 Predicted seminal fluid proteins transferred in *Aedes aegypti* ejaculate
(0.18 MB DOC)

Table S2 Predicted sperm proteins transferred in *Aedes aegypti* ejaculate
(0.11 MB DOC)

Table S3 Proteins identified from unlabeled unmated *Aedes aegypti* female sample^a^
(0.07 MB DOC)

Table S4 Amino acid sequences of unannotated predicted sperm and seminal fluid proteins from *Aedes aegypti*
(0.07 MB DOC)

Table S5 *Aedes aegypti* seminal fluid proteins and sperm proteins indistinguishable by peptides identified through mass spectrometry
(0.05 MB DOC)

Table S6 Putative *Aedes aegypti* sperm proteins that were not detected as transferred to females during mating
(0.08 MB DOC)
