# Supplementary material for: Towards a Semen Proteome of the Dengue Vector Mosquito: Protein Identification and Potential Functions
Source: PLoS Negl Trop Dis. 2011 Mar 15;5(3):e989. doi: 10.1371/journal.pntd.0000989 (PMC3057948; doi:10.1371/journal.pntd.0000989)
Supplement: Table S1 — Predicted seminal fluid proteins transferred in Aedes aegypti ejaculate (0.18 MB DOC) [file pntd.0000989.s001.doc]

Table S1: Predicted seminal fluid proteins transferred in *Aedes aegypti* ejaculate

| **Molecular function** | **Predicted protein class** | ***Aa****^a^* | **SV/TESTES^b^** | **M/S^c^** | **emPAI^d^** | | ***Cq^e^*** | ***Ag*** | ***Dm*** |
| --- | --- | --- | --- | --- | --- | --- | --- | --- | --- |
|  |  |  |  |  | TP1 | TP2 |  |  |  |
| **Binding** | Annexin | 11302 |  | M | 0.3 | 0.42 | CPIJ001340 | AGAP003790 | AnnIX |
|  | Calcyphosine | 08489 |  | M | N/A | 0.3 | CPIJ000193 | AGAP007666 | CG10126 |
|  | Dipeptidase | 08893 |  | M | 0.19 | 0.34 | CPIJ014907 | AGAP004775 | Dip-C |
|  | Fibrinogen | 01713 |  | M | 1.04 | 3.69 | CPIJ018551 | AGAP011307 | N |
|  | Kakapo | 02829 | Testes | M | 0.00 | 0.00 | CPIJ003239 | AGAP011396 | shot |
|  | Lectin | Supp4872 |  | S |  |  | N | N | N |
|  |  | 04679 |  | M | N/A | 0.74 | N | N | N |
|  | Mitochondrial brown fat uncoupling protein | 07046 |  | M | N/A | 0.19 | CPIJ019873 | AGAP011676 | Ucp4A |
|  | Moesin | 07915 |  | S | 0.05 | 0.05 | CPIJ012259 | AGAP000562 | Moe |
|  | Mucin | 00718 |  | M | N/A | 0.24 | CPIJ012373 | AGAP009473 | vir-1 |
|  | Odorant-binding | AaegSfp1 |  | S |  |  | N | N | N |
|  | Phosphatidylethanol- binding protein | 11263 |  | S | 0.31 | 0.31 | CPIJ008654 | AGAP002049 | N |
|  | Tubulin β-chain | 02848 |  | M | 0.68 | N/A | CPIJ003263 | AGAP10510 | BetaTub60D |
|  | None | 00479 |  | S | 0.02 | 0.02 | CPIJ016864 | AGAP001690 | Rim |
|  |  | 08274 |  | M | 0.1 | 0.33 | CPIJ020078 | N | N |
|  |  | 09201 |  | S | 0.35 | 0.35 | CPIJ017479 | AGAP012418 | CG7630 |
| **Oxidoreductase** | Catalase | 13407 |  | M | 0.12 | 0.12 | CPIJ007044 | AGAP004904 | Cat |
|  | Decarboxylase | 05790 |  | M | 1.75 | 1.63 | CPIJ012341 | AGAP000184 | Men |
|  | Dehydrogenase | 04338 | Testes | S | 0.09 | 0.18 | CPIJ016430 | AGAP010421 | CG11876 |
|  |  | 05308 |  | M | 0.24 | 0.24 | CPIJ004390 | AGAP004786 | N |
|  |  | 06928 | Testes | S | 0.06 | 0.13 | CPIJ019726 | AGAP011629 | CG7430 |
|  |  | 10464 | Testes | M | 3.29 | 4.28 | CPIJ014605 | AGAP004362 | Gdh |
|  |  | 12014 |  | M | 1.22 | 1.89 | CPIJ014454 | AGAP004880 | ImpL3 |
|  | NADH-ubiquinone oxidoreductase subunit | 05946 |  | M | 1.04 | N/A | CPIJ003877 | AGAP002889 | CG12400 |
|  | Peroxidase | 04112 | Testes | S | 0.33 | 0.33 | CPIJ003399 | AGAP011054 | Jafrac1 |
| **Proteolysis/ Catalysis** | Aminopeptidase | 02399 |  | M | 2.56 | 3.65 | CPIJ006613 | AGAP009685 | Got2 |
|  |  | 02978 |  | M | 9.64 | 8.5 | CPIJ009825 | AGAP003475 | Dip-B |
|  |  | 07201 |  | M | 0.15 | 0.18 | CPIJ011103 | AGAP003077 | CG8773 |
|  | Asparaginase | 02796 |  | M | 5.95 | 6.83 | CPIJ008683 | AGAP002397 | CG6428 |
|  | ATP synthase subunit | 06516 |  | S | 0.12 | 0.12 | CPIJ002428 | AGAP009486 | VhaSFD |
|  |  | 07777 |  | M | N/A | 0.16 | CPIJ015460 | AGAP003879 | CG8029 |
|  |  | 11025 |  | S | 0.27 | 0.27 | CPIJ009947 | AGAP000721 | VhaAC39 |
|  |  | 12035 | Testes | M | 0.13 | 0.28 | CPIJ006751 | AGAP002401 | Vha26 |
|  |  | 12819 |  | M | 0.25 | 0.57 | CPIJ006975 | AGAP001823 | Vha13 |
|  | ATPase | 14053 |  | M | N/A | 0.14 | CPIJ003274 | AGAP001587 | Vha100 |
|  | Dehydrogenase | 04294 | Testes | M | 0.27 | 0.19 | CPIJ016647 | AGAP007975 | CG5261 |
|  | Dynein | 11478 |  | M | 1.8 | 0.85 | CPIJ0156022 | AGAP001335 | Ctp |
|  | Gamma glutamyl transpeptidase | 10935 |  | M | 0.12 | 0.25 | CPIJ003186 | AGAP008915 | N |
|  |  | 14580 |  | M | 0.23 | N/A | N | N | N |
|  | Glutathione transferase | 11741 |  | M | 0.14 | 0.71 | CPIJ006160 | AGAP010404 | GstS1 |
|  | Hydrolase | 03666 |  | M | 0.58 | 1.63 | CPIJ011448 | AGAP009907 | CG10602 |
|  |  | 06485 |  | M | 0.09 | 0.39 | CPIJ012557 | AGAP001183 | N |
|  | Kinase | 12359 | Testes | M | 0.18 | 1.31 | CPIJ004571 | AGAP007120 | Awd |
|  |  | 12731 |  | M | 0.43 | 0.13 | CPIJ014541 | AGAP007722 | Adk2 |
|  | Lipase | 07063 |  | M | 0.56 | 0.43 | CPIJ004224 | AGAP011683 | N |
|  | Mannosidase | 05763 |  | M | 0.25 | 0.44 | CPIJ015656 | AGAP008584 | CG6206 |
|  | Protease | 01588 |  | M | 0.71 | 1.3 | CPIJ017373 | AGAP007619 | CG17337 |
|  |  | 02000 | SV | M | 0.82 | 0.7 | CPIJ002595 | N | N |
|  |  | 06403 | SV | M | 2.5 | 2.12 | N | N | N |
|  |  | 06414 |  | M | 0.94 | 0.39 | N | N | N |
|  |  | 06421 |  | S | 0.11 | 0.11 | N | N | N |
|  |  | 06429 |  | M | 1.4 | 1.67 | N | N | N |
|  |  | 10725 |  | M | 0.04 | 0.15 | CPIJ015181 | AGAP002915 | Fur2 |
|  |  | 11558 |  | M | 1.89 | 5.06 | N | N | CG15253 |
|  |  | 12217 |  | M | 0.13 | 0.13 | CPIJ014660 | AGAP000885 | SP1029 |
|  |  | 15386 |  | M | 0.98 | 0.98 | CPIJ005491 | AGAP008176 | CG11034 |
|  | Protease inhibitor | 02715 | SV | M | 0.25 | 0.16 | CPIJ007024 | N | N |
|  |  | AaegSfp2 |  | M | 0.25 | 0.93 | CPIJ018147 | AGAP006638 | N |
|  |  | AaegSfp3 |  | M |  |  | CPIJ010777 | AGAP007906 | CG31704 |
|  | Thioesterase | 03569 |  | M | N/A | 0.13 | CPIJ010432 | AGAP003848 | CG1635 |
|  | Transferase | 03746 | Testes |  | 0.2 | 0.06 | CPIJ003886 | AGAP004396 | CG7920 |
|  | None | 02793 |  | S | 0.05 | 0.05 | CPIJ015183 | N | N |
|  |  | 13559 |  | S | 0.01 | 0.01 | N | AGAP005471 | CG33519 |
|  |  | 17451 |  | M | 0.19 | N/A | CPIJ009106 | AGAP009751 | N |
|  |  | 17460 |  | M | 8.29 | 6.83 | CPIJ009107 | AGAP009754 | Ance |
| **Structural** | Actin | 01928 | SV | M | 0.25 | N/A | N | AGAP011516 | N |
|  |  | 05964 |  | M | N/A | 0.25 | N | N | N |
|  | Vitellogenin | 05815 |  | M | 0.11 | 0.24 | CPIJ016051 | AGAP005103 | CG5162 |
|  | None | 03348 |  | S | 0.04 | 0.04 | CPIJ004882 | AGAP008145 | CG9487 |
| **Transport** | Cytochrome c oxidase subunit | 00929 |  | S | 0.63 | 0.28 | CPIJ004219 | N | N |
|  |  | 13751 | Testes | M | 0.62 | N/A | CPIJ006093 | N | N |
|  | Glutamate receptor | 09813 |  | S | 0.03 | 0.03 | N | AGAP010272 | CG15627 |
|  | Mitochondrial glutamate carrier | 11276 | Testes | S | 0.1 | 0.1 | CPIJ017288 | AGAP010293 | CG18347 |
|  | Sodium/calcium exchanger | 12480 |  | S | 0.11 | 0.11 | CPIJ005951 | N | N |
| **Other** | Niemman-Pick Type C-2 | 09760 |  | M | 0.45 | 0.45 | CPIJ000782 | N | N |
|  | Rab GDP-dissociation inhibitor | 12904 |  | M | 0.07 | 0.14 | CPIJ015309 | AGAP010025 | Gdi |
|  | Venom allergen | 09239 |  | M | 4.99 | 3.85 | N | N | N |
|  | None | 04944 |  | S | 0.11 | 0.05 | N | N | N |
|  |  | 05219 |  | M | 0.57 | 0.57 | CPIJ011108 | AGAP013183 | N |
|  |  | 10824 |  | M | 0.89 | 13.46 | CPIJ016383 | N | N |
|  |  | Supp3543 |  | M |  |  | N | N | N |
|  |  | Supp4095 |  | M |  |  | N | N | N |
|  |  | AaegSfp4 |  | M | 0.63 | 1.07 | N | N | N |
|  |  | AaegSfp5 |  | M | 0.89 | 1.34 | N | N | N |
|  |  | AaegSfp6 |  | M | 0.45 | 0.64 | N | N | N |
|  |  | AaegSfp7 |  | M | 1 | 1.21 | N | N | N |
|  |  | AaegSfp8 |  | M | 0.28 | 1.35 | CPIJ016384 | N | N |
|  |  | AaegSfp9 |  | M |  |  | N | N | N |
|  |  | AaegSfp10 |  | S |  |  | N | N | N |
|  |  | AaegSfp11 |  | M | 0.6 | 0.27 | N | N | N |
|  |  | AaegSfp12 |  | M | 0.28 | 0.63 | N | N | N |
|  |  | AaegSfp13 |  | M | 0.48 | 1.41 | N | N | N |
|  |  | AaegSfp14 | SV | M |  |  | N | N | N |
|  |  |  |  |  |  |  |  |  |  |
|  |  |  |  |  |  |  |  |  |  |

^a^ 5-digit numbers are the Vectorbase database identification numbers without the proceeding “AAEL0”. Numbers with “Supp” prefix refer to proteins from the Supplementary predicted peptide database from AaegL1.1 Gene Build. Numbers with the prefix “AaegSp” refer to proteins from either the 6-frame translation or the small peptide databases. The amino acid sequences for all of the “Supp” and “AaegSp” predicted proteins are given in Table S4.

^b^ Protein was identified from either the sperm-enriched seminal vesicle (SV) or the testes sample (testes) and is hypothesized to be derived from that tissue.

^c^ M/S: The presence of multiple (M) peptide hits or single (S) peptide hits from multiple biological replicates to each protein are indicated.

^d^ emPAI is a measure of relative protein quantitation in a mixture. Ishihama, Y., et al. 2005. Exponentially modified protein abundance index (emPAI) for estimation of absolute protein amount in proteomics by the number of sequenced peptides per protein, *Molecular & Cellular Proteomics* 4: 1265-1272. TP: Transferred Proteins; SV: Sperm-enriched proteins from seminal vesicles; T: Sperm-enriched proteins from testes. We do not have emPAI scores for SUPP proteins and proteins from the small peptide database.

^e^ Homolog in the genome of each of the following species: *Cq: Culex quinquefasciatus; Ag: Anopheles gambiae; Dm: Drosophila melanogaster.* For the proteins from the Vectorbase database, we defined homologs as best reciprocal BLASTP hits with e-value ≤ 0.001 and identity ≥ 30%. For proteins from the other databases, we defined homologs as a unidirectional hit with e-value ≤ 0.001 and identity ≥ 30%. N: No homolog detected.
