# Supplementary material for: Towards a Semen Proteome of the Dengue Vector Mosquito: Protein Identification and Potential Functions
Source: PLoS Negl Trop Dis. 2011 Mar 15;5(3):e989. doi: 10.1371/journal.pntd.0000989 (PMC3057948; doi:10.1371/journal.pntd.0000989)
Supplement: Table S3 — Proteins identified from unlabeled unmated Aedes aegypti female samplea (0.07 MB DOC) [file pntd.0000989.s003.doc]

Table S3: Proteins identified from unlabeled unmated *Aedes aegypti* female sample^a^.

| Vectorbase Identification | M/S^b^ | Vectorbase Identification | M/S | Vectorbase Identification | M/S |
| --- | --- | --- | --- | --- | --- |
| AAEL000175-PA | S | AAEL005766-PA | M | AAEL009691-PA | M |
| AAEL000293-PA | M | AAEL005815-PA | S | AAEL009838-PA | M |
| AAEL000454-PA | S | AAEL005845-PA | S | AAEL010143-PA | S |
| AAEL000479-PA | S | AAEL005901-PB | S | AAEL010159-PA | S |
| AAEL000545-PA | M | AAEL005961-PA | M | AAEL010698-PA | S |
| AAEL000746-PA | M | AAEL005964-PA | M | AAEL010776-PA | M |
| AAEL000758-PA | M | AAEL006070-PA | M | AAEL010782-PA | M |
| AAEL001218-PA | M | AAEL006090-PA | S | AAEL010850-PA | S |
| AAEL001593-PA | S | AAEL006261-PA | S | AAEL010894-PA | S |
| AAEL001668-PA | M | AAEL006336-PA | S | AAEL011116-PA | M |
| AAEL001673-PA | M | AAEL006415-PA | S | AAEL011197-PA | M |
| AAEL001928-PA | M | AAEL006436-PA | S | AAEL011288-PA | S |
| AAEL002399-PA | S | AAEL006511-PA | M | AAEL011788-PA | S |
| AAEL002759-PA | M | AAEL006582-PB | M | AAEL011795-PA | M |
| AAEL002761-PA | S | AAEL006721-PB | M | AAEL011973-PA | M |
| AAEL002829-PA | S | AAEL006885-PA | M | AAEL012062-PA | M |
| AAEL002848-PA | S | AAEL006912-PA | S | AAEL012110-PA | S |
| AAEL002870-PA | S | AAEL006919-PA | M | AAEL012172-PA | S |
| AAEL002906-PA | M | AAEL007078-PA | M | AAEL012217-PA | M |
| AAEL002956-PA | M | AAEL007174-PA | S | AAEL012579-PA | S |
| AAEL003012-PA | M | AAEL007236-PA | S | AAEL012614-PA | S |
| AAEL003125-PA | S | AAEL007306-PA | M | AAEL012731-PA | M |
| AAEL003193-PA | S | AAEL007356-PA | S | AAEL012827-PA | M |
| AAEL003412-PA | S | AAEL007604-PA | S | AAEL012904-PA | S |
| AAEL003933-PA | S | AAEL007707-PA | S | AAEL012918-PA | S |
| AAEL004076-PA | S | AAEL007892-PA | S | AAEL013068-PA | S |
| AAEL004088-PA | M | AAEL007926-PA | S | AAEL013089-PA | S |
| AAEL004297-PA | M | AAEL008083-PA | S | AAEL013359-PA | S |
| AAEL004325-PA | S | AAEL008166-PA | S | AAEL013458-PA | M |
| AAEL004327-PA | M | AAEL008167-PA | S | AAEL013614-PA | M |
| AAEL004347-PA | S | AAEL008216-PA | M | AAEL014364-PA | S |
| AAEL004500-PA | M | AAEL008340-PA | S | AAEL014426-PA | M |
| AAEL004616-PA | M | AAEL008542-PA | S | AAEL014706-PA | S |
| AAEL004699-PA | M | AAEL008607-PA | M | AAEL015337-PA | M |
| AAEL004988-PA | M | AAEL008862-PA | M | AAEL016984-PA | M |
| AAEL005056-PA | S | AAEL009185-PA | M | AAEL017263-PA | M |
| AAEL005384-PA | S | AAEL009216-PA | M | AAEL017315-PA | M |
| AAEL005524-PA | S | AAEL009389-PA | M |  |  |
| AAEL005656-PA | S | AAEL009583-PA | M |  |  |

^a^ Samples are from proteins in molecular weight ranges ~30kD to 50kD and ~98kD to 120kD.

^b^ M/S: The presence of multiple (M) or single (S) peptide hits to each protein are indicated.
