# Supplementary material for: Towards a Semen Proteome of the Dengue Vector Mosquito: Protein Identification and Potential Functions
Source: PLoS Negl Trop Dis. 2011 Mar 15;5(3):e989. doi: 10.1371/journal.pntd.0000989 (PMC3057948; doi:10.1371/journal.pntd.0000989)
Supplement: Table S4 — Amino acid sequences of unannotated predicted sperm and seminal fluid proteins from Aedes aegypti (0.07 MB DOC) [file pntd.0000989.s004.doc]

Table S4: Amino acid sequences of unannotated predicted sperm and seminal fluid proteins from *Aedes aegypti.*

| **Protein Identification^a^** | **Database^b^** | **Amino Acid Sequence** |
| --- | --- | --- |
| AaegSp1 | 6-frame | MISKLLTLTALLVFTHVRATFVVYPPKYELSDAQTEELMACLTPTENVGDQQSKYDPICGTDGFSYYNKYQMKCMAKAIPSVDFAFYGRCPNEPMFLPLESARGTELLGNQAEFDKFQRCLESCNAFSPVCGNDQKTYSSPCALQCAKAHRPKLDVKASGFCKEDQTVDDKCPEILQPFCGSDGITYLNYCQFKFAQLGEKDLLPAHMGDCVRRLIAVKGALQKKSGGCGCGGGGGCGGCGNPWGDAVVMAKTKGAIAPQEKSPELLRSIIDVLFLHHPISPWPPAPYAAEEVARVTNEELAEVVKSFASNKAPGPDGIPNVAIKAAVNADPDMFRTTMQRCIDQGIFPEATEIGATTKGGETTRLVPYTKSADGLSNNQFGFRKVGIRYCAVATLDVKNAFNSASWEAIAHSLHRLKVPVQLCKLLESYFDGRILLYDTEEGQKSVLITARVPQGSILGPLLWNAMYDDVLRLPLPTGIKIVGFADDIFPVVYGESMEEVELTAAHSISIVEEWKKSRKLGLARHKTEVVVVNNRKCEQRVLTSVGDCTIEFKRPLRHVEVMINDTMNMPVKGHPRQRFGG |
| AaegSp2 | 6-frame | MTNFVPHNSAFRIEKIPDFKEPLYLIRLINVQEFVFGGWTNTVTHYNRRPVFSYVYDYNGTQGDVDIWMFEKVIKNGKVLGSWYIRNAHYGEYMIASTKSSEPPPNAGPAAQFHTVKLDRTPQPDLTIEHQFFFEDC |
| AaegSp3 | 6-frame | MADAQSNCGASNDYGMAMQAATPNDVRTQPTRYCYAHGPLLPTSLLPKPRKESYVDIGSVSCEEVVGYLMGLNRWYDRKCSNMGPECPDKYRFAATAEVDPEQSCIPGTIGSIKSNLMRTLIICYVYGCPEEVTTSIAANFNQFHNADLYPNNDTTAAAATAPKTPVQEVPLKILSKLAKLTSAMEKDRTAALTACTDYVSLLNKLVVDVLQIKSKCYLEPQAQYPPETTEDGGAIDCDEMTVYMKQMKKYFSTQCDSKGKNCPDKLKSAVSWFSRAYKQMGKSCYYDAYEYLDR |
| AaegSp4 | 6-frame | MYYCQITPNALATVDRTGWNSRVDHALNEIIRGHHGYKPDQSIYGDINRMFDATLCTVILIASGDRNISEEHLLWSEAFLHGIRDRYGKHPPNVRKMLGFIKHIQGELAAGKDSSMTEKMPMADGFASMVPTAKNKKLRSRN |
| AaegSfp1 | Small peptide | MILFAICSPLFHLSGAVRFTEEDYRVFRQKCSKILQTSSGTVEKAEQKNFHTDSEEMNCLIRCVGIMSGFYDDETGTNWDLVREQLNDKDGFDEHQAATTACTDALPEEELTSSVCRKSYLFFRCAMKSAKEHIREKS |
| AaegSfp2 | 6-frame | MSASKLTLTLLILVVALVALASAQWPPVKSGPGDKRCYCFRTFTPVCGTDNKNYLSLCHLQCAKHFYEVDVKSNGLCPGGNDNIVLGDIDPSAFPWSG |
| AaegSfp3 | Small peptide | MGSISLSLLILLVVTTTTLVIEGAPPSPPPRKNLTVQDELNRFYTLLSTLSHHSNRSIKETICMVPMILEPVCGSDGQTYGNQWLLECSAKRADTKLTVAKKGKC |
| AaegSfp4 | 6-frame | LSFNSCTCPCRGLVAVQVNPYLKTNANCVYIKSVYSKGFLFTSDVVIDGDRYVFHDVSHYKTAKPNYSHTAFWKIFYPWKNVSDTADVPLAVQNILSTEYLVVSHKIVQFTNREIVTHPVLTKYSLWHFTYPGQYYKLQNQFTKEYLFSDEQHRSGWNEGKVFTDTIKRSSSDDFPGKYGFYIMPCYE |
| AaegSfp5 | 6-frame | MEDGGHLAYVDKYRPPSGTLVSTLRIKHTAYWKLYYPWKNGTGDVPKGFLFTSDVVIDGDRYVFHDVSHYKTAKPNYSHTAFWKIFYPWKNVSDTADVPLAVQNILSTEYLVVSHKIVQFTNREIVTHPVLTKYSLWHFTYPGQYYKLQNQFTKEYLFSDEQHRSGWNEGKVFTDTIKRSSSDDFPGKYGFYIMPCYE |
| AaegSfp6 | 6-frame | MYDAAMWEATTTDPQSHGINIMNIRTTSFMYASSNADSMSPACRNLFLRSDARETQGDASRFLFHYNSTTKAYRIQNLVTKEFVQNLPSAFQDNLAIVCLDRRGGVERGAEENYDFWLKSCDDEYFENKKITAEINSQFG |
| AaegSfp7 | 6-frame | MNNPVLPILLAIVALSTICNAYRIGPSNAVPPRSLSSSYSNGNLLDSIRNIAGSIRWDQVLQDAFDRFESREPVAPIIVRLPGPIPKGQLREVVRRLEALFNDLWGQSTSEIEDVGPRVNTDALVKGIKEVMEKEKEAKEEVVSDMTNVKSVLKEVDDATKQSELLKEVGDYLNNKTEESSQKMNDREQSVKQPPPLEDEPIEQPVSSTTTEVPDSRSGSNVDEMEPPPNPIAGISAILKEEARKLTEKEPEKSSEEQLKEQVQQLKASLKDLVGVIDQIIESGAVSKSLVDQVDEVIPEALVAADEIASQIGAKT |
| AaegSfp8 | 6-frame | MGLRYELFFLLVGIFPDIGYTEKPSASFCQCPCTNRVAINKDWFNCMTIYSVRTGGYLTADEHYPFLKYYERYVFVTNKNKLWQTAKWKVVYKDANDRTYSLKNEYVKEWFHAGIDEQARDAYRRYALTKIRGDENVPPKDAYWQFIPDPKIGKDVYRIRNAFTDEYLYVDDEQHSQRVWWGNESASQSKAGGRDR |
| AaegSfp9 | Small peptide | MDFYRVLAVFESNIVPRNSAFRIERIREYEEPLYFIKLISSREYVFGGDMSTSGDTYRLPVFSYHHRVNTTRKDDGIWGFEKAYVNRVWRGAWYIKNVYYDNYMIASTVHADPPPMAANARFKTLKLDRRRHIPLTTEHMFYIEEC |
| AaegSfp10 | Small peptide | MQLATAQSLDDYNELGRLLNQQQYKEQDFWATDQDIDRNIAWSFTFNEAEMTLIGQEGEFLPRSFAI |
| AaegSfp11 | 6-frame | MEYISAINDDETVLDLKRGIMISHPAVMQDQDPVQDAQNTTCTSCRLNVDKRAICTSSVDQVAGSSKTTTTEELLDAPTTTEELPEVPSADSLATVPSATSVSTNQSEDECIQKVNIERFNEGIAGIKVTPIKWTKMGYVNYPEKKYREINEAVRRNLFKLGPEDVENTDYDEHEKVIYPRYGQTATAMKNLPLIFLMLCIAVKIHLSESFPQCCPMMCCSCGCGSGQGSSSDFSPPGDDPNDGQADDGLVRKAGKRHRWEKADTIISGVQAVADVGNLVKDVAQAVSSSGDHGS |
| AaegSfp12 | Small peptide | MSSMKESIVELMFLASKIGNPGDRVRVTEWIRKLSETAAEPDAHPRLVEEYMDYLKLLLSSSPIYFVNPFKSYPPKNHRLVPLAESLGNSLANECPYLPRSGPLKPILLHRSEDDTAVISVHQNQQGEVMCYMAITPRNE |
| AaegSfp13 | 6-frame | MSVLRAKYCYEHGWMLAAIVGSDEERRVTALAQRNNPTGWLNPRFWVAENDQEEDAEFCPQITLKNYMEGANDCMEDLYFVCESIE |
| AaegSfp14 | 6-frame | MAFSQSLQCKACDSEISWDDCQSKIFLEDCGLIISPNKPNHVHQCFQLEENDPTTKKTLYRQGCTGDVAFCSERPKENMQQCSLCTDDSVSSECTSMVHRVKKTSDGKLVDVVDTIHFQRTEAKANPSESGAPPVNSSTVIDFASKKNGSDEANTLKVEMKNGQTSEKDETGVQGTNQTEGALSSTEPPTIVTLKTASITPPKSACNRNVKTPATSTEKDQGIYTIQPDGGNATETSTSSSVDPQGPMIATGPPEVKSSRSFGLANRAAPIWVVGLLVVLNFRNVDM |
| SUPP0622 |  | MGPSGIIVVLFVVYLVVADGSPCCQSCCACCSGCGGGCDGGSMRQADPYAGQASANFEQVMRKVIGKMAKNKTVKPSENAIPDYVEQPADSEVRAAKPGAAKPAAANKPFDGVGPIRYENDPGDPPPKMDPPPMAPSVGKVVEPVYWLD |
| SUPP2074 |  | MSNGRQTIDDSEPLAGIINTIDTLENTLSNLELDVRNELNAQRLLYRCQLNLAGRCPSVDRVRDTDSQTSQGATSIVSKYSACNCRCNQALEIYLAQLRKAQLDQEELLRTMKMKDEQAKLYRCKLMESNALVERQKQEIKALKDNEELITQKINTALEEENQTLMTEIERLKSLPDELRARERALKLANKELQETKLTLKSLLLDIESGLETCEDISGELQQERKRAFHTLNEIDEEKRKVLSWIAKYSELKQQYDTAVQQKESVAQLSAALREKTSKLDALTKDYDALKKESVDYISNVETVNEKQRTALQERVVELECQNLQYKIALEEQCQKTSEVSHNMQRELLNLEMKFVEAQEEVNAMKIYNEKAAASAEYTARRKSLVGDETSMDSQSLSPPYCKTCGVEFSSENVDSEHSCSKDILPSKSENNDTATRSIKSEENDTKSKSMESNS |
| SUPP3540 |  | MGVVPPIGRRKKSKSTDLLKSGETSGNVQFSVASWRPLVSIRAVTFFGLVKMCLRSVSDRNQLILANCCCSVGGGAVE |
| SUPP4095 |  | MNNPVLPILLAIVALSTICNAYRIGPSNAVPPRSLSSSYSNGNLLDSIRNIAGSIRWDQVLQDAFDRFESREPVAPIIVRLPGPIPKGQLREVVRRLEALFNDLWGQSTSEIEDVGPRVNTDALVKGIKEVMEKEKEAKEEVVSDMTNVKSVLKEVDDATKQSELLKEVGDYLNNKTEESSQKMNDREQSVKQPPPLEDEPIEQPVSSTTTEVPDSRSGSNVDEMEPPPNPIAGISAILKEEARKLTEKEPEKSSEEQLKEQVQQLKASLKDLVGVIDQIIESGAVSKSLVDQVDEVIPEALVAADEIASQIGAKT |
| SUPP4104 |  | MCNPFVVLILLVATSYSASAIYPFGLGGRQPQGYLNVVFPARPQYRYYEIARNRHLLEAIRHIVMAGSLRQDEVMRKASVRLRPWTPSNRRLPKVPPVVMPMVGSSASPTKISIPIPPPVPAVISEKTADVSGKEPTKEVIALAPESKEKIKSVINELEENMEQDIQKAADDVGSVAPPKVTQEVIEKTIAKVEEIVHQSGDDSDLRTEMQTEAPALNVAVSAVPDAVVLVDVAEKQETPKEETPKEETPKEETPTIMDSPEKPSESKPEKSGTETLKKLITQLGSSLTKLMGTMDQLVKTG |
| SUPP4130 |  | MAKAPAVGIDLGTTYSCVGVFQHGKVEIIANDQGNRTTPSYVAFTDTERLIGDAAKNQVAMNPTNTIFDAKRLIGRKFDDPAIQADMKHWPFDVISVEGKPKIQVEYKGETKNFFPEEISSMVLTKMKETAEAYLGKTVSNAVVTVPAYFNDSQRQATKDAGTISGLNVLRIINEPTAAAIAYGLDKKTAGERNVLIFDLGGGTFDVSILSIDDGIFEVKSTAGDTHLGGEDFDNRLVNHFAQEFKRKHKKDLSTNKRALRRLRTACERAKRTLSSSTQASIEIDSLFEGTDFYTSITRARFEELNADLFRSTMEPVEKAIRDAKMDKASIHDIVLVGGSTRIPKVQKLLQDFFNGKELNKSINPDEAVAYGAAVQAAILHGDKSEEVQDLLLLDVTPLSLGIETAGGVMSVLIKRNTTIPTKQTQTFTTYSDNQPGVLIQVFEGERAMTKDNNLLGKFELSGIPPAPRGVPQIEVTFDIDANGILNVTALEKSTNKENKITITNDKGRLSKEDIERMVNEAEKYRSEDEKQKETISAKNALESYCFNMKATMEDDKLKDKITDSDKTLIMDKCNDTIKWLDANQLAEKEEYEHRQKELESVCNPIITKLYQSAGGAPGGMPGFPGGAPGAGAGAAPGAGSGSGPTIEEVD |
| SUPP4499 |  | MDWLPLHSGCPKCGFKNPATCHNDAPSTLHETTPNRLNSWMGSLELPEPQLQRPSTGTLRSTSLNEMMKPCPICRLRGGCCPDCKKRASTAQNHRDPNSTTQSSTSDSEMHPRRVMERPKTRPSLRDRFNIFPKKIDKATRLSDLYKAYGDKDTNPHEVSQRRSTKSSIVSLNVEEILKKTDTHKRKITSGGIQSVELRKVQSSREADRNNKTVEKCDSPTHAQIRKNQRSLLQRIKKQNRGKYSYRYGQRYPGIVIGHRECIQQGRQVPPHMGWMWNVKTLGINKIRKGWRPGAVKKPIKELMQHFLVSYPLDNIPVSKKTGRNLKIPADGSEHTKQKPTLQIVKKNGEYCIVMNPLKDSASLKTAQDPYLNCEPIRFKLAKDPNVGKLYQLRSALKVKGFTMCGCSELESCEHKSEKEKKLLRKELRKLAKCLGLPKSTELKDVPIDSESELDLEFTPPSAMLKSGQRKPDVVCTETQYSVDDYKVQVPADKLKCKPGREDPNDVGKGLKGKGGQVRDKAGKDGKGGKGGKDGKGQGKGGAGSGVGAGAGGKAKTGPSSKAGVGGAKAGAAGTTNK |
| SUPP4501 |  | MEQPSISSATGSNISDVVVQDPDRNGSIQKESYSLVIVLKSLVLANVENDDPRTLDISLLLGETEAKLNGTLADFNGQRHGTAISVPTSNSEEFKRYLTSHELLITIGTDLEVLGQTNIPLAATNLASFDPPAFEPIIINDAFPIIHESQPIGKIDLALKTDRTKSPPKPDGPKDGPEEEDSMLYIVNDASPRQSNEFQDDIMRQLLTCKKCNALRSPSEMSYQYELIDGILVNKEYPRKDPDLETMKRKIEQIEWEAKLYPVGKEQPPPKEPAHHRFCDGCGGYSITGATCTNRMQAVDVPLSEAQFRYPEGSAKR |
| SUPP4872 |  | MYLLHVFTVTLSLLLLLPSEVISKSRSYVFPNGLVTFEEAWANCKDKGMQLATAQSLDDYNELGRLLNQQQYKEQDFWATDQDIDRNIAWSFTFNEAEMTLIGQEGEWAKTRCVLVRSFFHAGTAGTDWNDDLCGQRHRYICDRL |
| SUPP7141 |  | MNPEFQEIVSPRQLRHSPVRDSDAHYRQQNSSQTRGLRTPGFTSRIPPPSATGRRQPTQGLITPSRIRPPSGSQLQTPQAIRRPEQIRPPSGLRPPGFFKPSLQSTPLAASSSSQKVIPLPGSANVLAQVPRPSRIPADPTSSTRIAAPPVSRVPSTSLLPKPSFLRSQSQSRIVPPSKRTSTPQRLTTPSQSRLVRSAPRATGRPRTAPATPRAVRRVEPSTPVRRPQAQLPRTAVKQRIVTDDAEVYSIAGREVEFVDWVPSPEEGPSRQPVRQRPTSRSGKVQIAKGIAKKAPSRGAIPVEPKTPVGKARIHLPRTAAKQRVSTEDDDAEVFNIGGRVVEFVDYVPTPEEKPEPEPIRRPVARRGAARIRMTASSDGSPEGATLPPPIVEAMQAMKKSRKVHEFPDEPSVAAAKEFASKIDADIQRFRGMGADMAELKQRKSAVLSKIRSMYDKETHEDAVVLDVEGFRTKYGVTQSPKMRQLLEVTSDWNRAILDRQELAESLAQAKSFVPQEKPKLTTMLQGLAESPDRPDQVTQKKSELELARQERIDNYRQKKEADREYRRILEERRKNLPSRRPYSKQEREDIRKRYQEAQEWTYTAPTEEELDDALRLAEMMELEDVGVSVVADPEKTVETLFPEAAPSAEISGVVGDVPIPRNIPAEEINNMNNLDRTTAEVALKASLVPVPTEGVTKQVLETSIKEMETAKIEPTPQKLDPVFEIDLKLGQPKRRNYIQLQGSSFPSWFPGFYSIFPGAPGLIKAMKSPSVTLLTAFRQMHSSAAI |
| SUPP8209 |  | MQTSSTTCDFEKDIVQVLAKVKGQVVRRKLRIIDKMQGFDPLNHFRITKNQFERGLSTANINVTPAEVCTLVNMFRTPLQDTVDYKRFCDTIAEIDYQSKLEKAPLLVPLKHFPSENGILNHLNFEERTIASRTLQKLARYADVVSNLSSLLEDFDRNRMGLVNRNQFIRALATRDLHTAISSREFDVLCKCFGVELGHRREINYRALLMALDYLYANKENHPF |
| SUPP8260 |  | MYKILSEAAKGISVKHLEALGKLAKVRPSSSSSTSKGHGLTDFSKKKACKEKVCRQRPPQPPLEKKPESYSETSKTWHTCPEPAKPKKFSCDDIQPQRVPRRSKLQVATKTPCMEPPPTLQAPDCFKVKTQLCPRTSMPGCGKARIPPRCEPKKVARDCIRAKPPFPSFSECFKFPFLPPPRSECTCLTEKKICQ |
| SUPP12054 |  | MEIRLILILTIVVSLDGISGAPCGGCCCIQTCCCSCSEPEADLEYSSEQVAETRSSNVFNNLWYNVGKAVGQSVWSPNGAAQILSGGASPGMLPTQGLPNGPTGAVNPFGPSGGTISSAQPQLQPGYGQSIGQQSQLEQQPQLFQPPNPQLTLEQHPEYVQGQNPQSSQGSVPPEVLEPEPQMVQQPEPQVSPQQETQLVQQPDPQFSQQTESQLGQNSNQETTLQTMQPPNQQPGLQQQPGQYMQQPAQQAGQQPGQLPMQPSMQLPMQPPMQQPMQRPMQQPGQQFLQQQPLVQYPQQFIQQAGQQYLQLPGQQNVQQWPGQYPTQQQPMQQPGQQYAPQHQQRPMQQYGQQYGQQYGQQYGQQYGQQPYQLPAAPYQAPGQYNPYHQNPTNPYKTKNSKKFIRDTYYHPPQ |
| SUPP12070 |  | MDSDPGVVLVEKVEEDEISTDKDSGMESAGNSKDDTPERKTATLEDLLQDDEMDPIVAAPAPSAPPAPAQQPAAPEVAAAPAPATQKPQPTASSTASKASAPAAIKSKDDDYDDEPDETLGERLWGLTEMFPETVRNVTGAVTNFSVASVKTVYKLTCNASWIFFTSSMILFAPIVFEVERAQMEEMQKSQQKQVLLGPGSAVGGGGPGGMPAMPPMAAR |
| SUPP14650 |  | MRCVQSIISRISSALPFLFRASATANRKRAHSLLPVVVLPLECHKHPSQFRAFLQENFKSMERDAVCPPCPSSPRERSSGANGMTGKPRGKVTTNPFFNFLRDYRGKHPNLSVVDAAVEGANVWNRMGPEERAPYVKQACGHPQRVTPCGVQSPRSGSRKRSRASSRGRRVSRSASRRGRKRNRSSSSGGRSRSRSRSKARRARRC |

^a^ AaegSp: Predicted sperm proteins; AaegSfp: Predicted seminal fluid proteins; Supp: Proteins from the supplemental predicted peptides of the AaegL 1.1 Gene Build

^b^ 6-frame: 6-frame translation of the *Aedes* genome (versionAaegL1.2); small peptide: database of small (<150 amino acid) predicted peptides
