# Supplementary material for: Towards a Semen Proteome of the Dengue Vector Mosquito: Protein Identification and Potential Functions
Source: PLoS Negl Trop Dis. 2011 Mar 15;5(3):e989. doi: 10.1371/journal.pntd.0000989 (PMC3057948; doi:10.1371/journal.pntd.0000989)
Supplement: Table S5 — Aedes aegypti seminal fluid proteins and sperm proteins indistinguishable by peptides identified through mass spectrometry (0.05 MB DOC) [file pntd.0000989.s005.doc]

Table S5: *Ae. aegypti* seminal fluid protein and sperm proteins indistinguishable by peptides identified through mass spectrometry.

|  | **Protein prediction** | **Gene 1** | **Location**  **(SuperContig; start location)** | **Gene 2** | **Location** | **Gene 3** | **Location** | **Gene 4** | **Location** |
| --- | --- | --- | --- | --- | --- | --- | --- | --- | --- |
| **Sfp genes** | **Lipase** | AAEL005815 | 1.175;  13,308 | AAEL012311 | 1.677; 316,677 |  |  |  |  |
|  | **Metalloproteinase** | AAEL011558 | 1.589; 624,565 | AAEL013449 | 1.847; 211,917 |  |  |  |  |
|  | **Pyruvate dehydrogenase** | AAEL005308 | 1.150; 852,728 | AAEL014865 | 1.132; 2,537 |  |  |  |  |
| **Sperm genes** | **ATP synthase beta subunit** | AAEL002827 | 1.69;  133,095 | AAEL003393 | 1.85; 595,731 |  |  |  |  |
|  | **Cytochrome C oxidase subunit** | AAEL005170 | 1.144;  81,906 | AAEL013009 | 1.775; 455,022 |  |  |  |  |
|  | **Histone H2B** | AAEL015674 | 1.9; 3,650,652 | AAEL015676 | 1.9; 3,675,254 | AAEL015677 | 1.9; 3,692,611 | AAEL015675 | 1.9; 3,665,929 |
|  |  | Cont. |  | AAEL015680 | 1.98; 809,318 | AAEL015679 | 1.98; 765,782 | AAEL015678 | 1.98; 751,208 |
|  |  | Cont. |  | AAEL015681 | 1.98; 871,838 |  |  |  |  |
|  |  |  |  |  |  |  |  |  |  |
|  | **Histone H4** | AAEL000490 | 1.9; 3,668,611 | AAEL003838 | 1.98; 794,216 | AAEL003866 | 1.98; 801,811 | AAEL000513 | 1.9; 3,677,938 |
|  |  | Cont. |  | AAEL000501 | 1.9; 3,695,307 | AAEL003814 | 1.98; 727,922 | AAEL00517 | 1.9; 3,658, 697 |
|  |  | Cont. |  | AAEL013709 | 1.901; 22,119 |  |  |  |  |
|  | **Mitochondrial aconitase** | AAEL003734 | 1.95; 2,765,510 | AAEL012897 | 1.756; 369,702 |  |  |  |  |
|  | **Prohibitin** | AAEL012882 | 1.675; 317,012 | AAEL013952 | 1.967; 11,182 |  |  |  |  |
