# Supplementary material for: Towards a Semen Proteome of the Dengue Vector Mosquito: Protein Identification and Potential Functions
Source: PLoS Negl Trop Dis. 2011 Mar 15;5(3):e989. doi: 10.1371/journal.pntd.0000989 (PMC3057948; doi:10.1371/journal.pntd.0000989)
Supplement: Table S6 — Putative Aedes aegypti sperm proteins that were not detected as transferred to females during mating (0.08 MB DOC) [file pntd.0000989.s006.doc]

Table S6: Putative *Aedes aegypti* sperm proteins that were not detected as transferred to females during mating.

| **Identification^a^** | **M/S^b^** | ***Dmel* homolog^c^** | **Identification** | **M/S** | ***Dmel* homolog** |
| --- | --- | --- | --- | --- | --- |
| AAEL000494-PA | M |  | AAEL006834-PA | M | CG7470 |
| AAEL001073-PA | M |  | AAEL008428-PA | M |  |
| AAEL002759-PA | M | FBgn0004117 | AAEL009345-PA | M | lethal (2) 37Cc/CG10691 |
| AAEL002764-PA | M | FBgn0037891**^d^** | AAEL010608-PA | M |  |
| AAEL002878-PA | M |  | AAEL011184-PA | M |  |
| AAEL002956-PA | M |  | AAEL011406-PA | M |  |
| AAEL003164-PA | M |  | AAEL011746-PA | M | skpA associated protein/ CG11963**^d^** |
| AAEL003415-PA | M |  | AAEL011871-PA | M |  |
| AAEL003501-PA | M | CG10252**^d^** | AAEL012062-PA | M | Sodium pump α subunit/ CG5670 |
| AAEL004366-PA | M |  | AAEL012950-PA | M | CG6343**^d^** |
| AAEL004423-PA | M |  | AAEL016984-PA | M | CG12055 |
| AAEL004616-PA | M |  | AAEL017395-PA | M | CG1907 |
| AAEL004631-PA | M |  | AAEL017511-PA | M |  |
| AAEL004659-PA | M | CG10659 | AaegSp4 |  |  |
| AAEL004872-PA | M | CG17349**^d^** | Supp0622 |  |  |
| AAEL005084-PA | M |  | Supp2074 |  |  |
| AAEL005108-PA | M |  | Supp2823 |  |  |
| AAEL005422-PA | M |  | Supp4499 |  |  |
| AAEL005435-PA | M | CG3731**^d^** | Supp4501 |  |  |
| AAEL005656-PA | M |  | Supp5151 |  |  |
| AAEL005733-PA | M |  | Supp8209 |  | CG10841**^d^** |
| AAEL005961-PA | M |  | Supp8260 |  |  |
| AAEL005989-PA | M |  | Supp12054 |  |  |
| AAEL005991-PA | M | Scheggia/ CG6782 | Supp12070 |  |  |
| AAEL006017-PA | M |  | Supp14650 |  |  |
| AAEL006721-PA | M |  |  |  |  |
|  |  |  |  |  |  |

^a^ Numbers starting with “AAEL” are the Vectorbase database identification numbers. Numbers with “Supp” prefix refer to proteins from the Supplementary predicted peptide database from AaegL1.1 Gene Build. Numbers with the prefix “AaegSp” refer to proteins from either the 6-frame translation or the small peptide databases. The amino acid sequences for all of the “Supp” and “AaegSp” predicted proteins are given in Supplementary Table 4.

^b^ M/S: The presence of multiple (M) or single (S) peptide hits to each protein are indicated.

^c^ *Drosophila melanogaster* homolog to *Ae. aegypti* protein*.* For the proteins from the Vectorbase database, we defined homologs as best reciprocal BLASTP hits with e-value ≤ 0.001 and identity ≥ 30%. For proteins from the other databases, we defined homologs as a unidirectional hit with e-value ≤ 0.001 and identity ≥ 30%.

^d^ The *D. melanogaster* homolog is a sperm protein: *Dorus et al.* 2006.
